# Long-Lasting Alterations in Gene Expression of Postsynaptic Density 95 and Inotropic Glutamatergic Receptor Subunit in the Mesocorticolimbic System of Rat Offspring Born to Morphine-Addicted Mothers

**DOI:** 10.1155/2018/5437092

**Published:** 2018-06-10

**Authors:** Pei-Ling Wu, Yung-Ning Yang, Jau-Ling Suen, Yu-Chen S. H. Yang, Chun-Hwa Yang, San-Nan Yang

**Affiliations:** ^1^Department of Pediatrics, E-DA Hospital, Kaohsiung, Taiwan; ^2^School of Medicine, I-Shou University, Kaohsiung, Taiwan; ^3^Graduate Institute of Medicine, College of Medicine, Kaohsiung Medical University, Kaohsiung, Taiwan; ^4^Research Center for Environmental Medicine, Kaohsiung Medical University, Kaohsiung, Taiwan; ^5^Department of Medical Research, Kaohsiung Medical University Hospital, Kaohsiung, Taiwan; ^6^Joint Biobank, Office of Human Research, Taipei Medical University, Taipei, Taiwan

## Abstract

Prenatal exposure to morphine causes altered glutamatergic neurotransmission, which plays an important pathophysiological role for neurobiological basis of opiate-mediated behaviors in such offspring. However, it is still not clear whether such alteration involves gene expression of ionotropic glutamate receptor subunits. In this study, we further studied whether prenatal morphine exposure resulted in long-term changes in the gene expression of *α*-amino-3-hydroxy-5-methyl-4-isoxazolepropionic acid (AMPA) receptor, *N*-methyl-d-aspartate (NMDA) receptor, and postsynaptic density 95 in the mesocorticolimbic area (an essential integration circuitry for drug craving behavior), nucleus accumbens (NAc), ventral tegmental area (VTA), and prefrontal cortex (PFC), of rat offspring from morphine-addicted mothers. Experimental results showed that prenatal morphine exposure led to a persistent downregulation of gene expression in the AMPA and NMDA receptor subunit, with a differential manner of decreased magnitudes, at the age of postnatal days 14 (P14) and P30. However, in PFC, the gene expression of the AMPA receptor subunit was not synchronized in observed rat offspring subjected to prenatal morphine exposure. An upregulation of gene expression in the AMPA receptor subunit 3 (GluR3) was persistently observed at P14 and P30. Furthermore, the gene expressions of PSD-95 in NAc, VTA, and PFC were all decreased concurrently. Collectively, the results suggest that prenatal exposure to morphine may initiate molecular mechanisms leading to a long-lasting, differential alteration in gene expression of the inotropic glutamate receptor subunit and PSD-95 in the mesocorticolimbic circuitry in rat offspring. This study raises a possibility in which differential changes in gene expression with a long-lasting manner may play a role for the development of nearly permanent changes in opiate-mediated behaviors, at least in part for the neurobiological pathogenesis in offspring.

## 1. Introduction

Maternal drug addiction is a worldwide growing issue; particularly, it is associated with increased perinatal mortality and morbidity in children. Using morphine in pregnancy might cause spontaneous abortion, preterm delivery, multiple congenital anomaly, and neonatal abstinence syndrome [1]. Neonatal abstinence syndrome is a complex disorder that mainly involved the central and autonomous nervous system and gastrointestinal system. Neonatal abstinence syndrome might present symptoms with mild irritable tremors, high pitch crying, fever, and seizure [1]. However, prenatal drug exposure not only causes perinatal effect but also might lead to long-term cognitive impairment and increased prevalence of drug addicting, craving behavior in the adulthood [2–4]. Although environment factors might be important for developing later neurological negative consequences, the alternated function and structure of central nervous system in these prenatal-drug-exposure neonates might also play an important role.

The synaptic plasticity of central nervous system during the early stages of life was easily affected. Exposure to multiple abusing drugs such as morphine, heroin, cocaine, and amphetamine exerts long-lasting changes in glutamate receptor-mediated synaptic networks, therefore leading to maladaptive alternations in gene expression and behavior adaption [5–7]. Indeed, opiates agent can cross placenta to affect the development of the central nervous system and lead to neurobiological behavior alternations in young offspring. However, little is known about the underlying molecular mechanisms involved in such alterations.

It has been believed that the mesolimbic dopamine pathway is involved in drug addiction and the glutamate systems, especially *α*-amino-3-hydroxy-5-methyl-4-isoxazolepropionic acid receptor (AMPAR) and* N*-methyl-d-aspartate receptor (NMDAR), play an important role in opiate addiction and craving [6]. And the postsynaptic architecture of postsynaptic density protein 95 (PSD-95) with glutamatergic NMDAR-mediated transmission could also play an important role for drug (e.g., cocaine)-dependent synaptic plasticity [8]. Previously, we have demonstrated that prenatal morphine exposure alters the kinetics mechanism of the NMDAR in the CA1 pyramidal neurons in hippocampus, which is an required integrated area for learning and memory, with altered synaptic plasticity in rat juvenile offspring (postnatal day 14 (P14)) [9, 10].

Thus, to advance our understanding of the mesolimbic circuit-related adaptations to prenatal repeated opioids exposure, we used a prenatal-morphine-exposure rat model to investigate the possible changes of long-lasting impact of morphine on the gene expression of the AMPAR subunit, NMDAR subunit, and PSD-95 in nucleus accumbens (NAc), ventral tegmental area (VTA), and prefrontal cortex (PFC) of offspring from morphine-addicted mother.

## 2. Method

### 2.1. Animal Model

Adult female Sprague-Dawley rats were separated arbitrarily into four groups and were injected subcutaneously twice a day as follows: vehicle-control and morphine hydrochloride. Morphine was gradually increased at the rate of 1 mg/kg at 7-day intervals from a starting dose of 2 mg/kg, as previously described [9–12]. Experimental animals were mated between days 7 and 8 and drugs were continued during pregnancy. After rat offspring were born, the doses of morphine administrated into the maternal rats were augmented 1 mg/kg every two weeks till postnatal days 30 (P30). And the offspring were sacrificed at P14 and P30 after birth.

### 2.2. Slice Preparation

Brain slices were prepared from rat offspring from morphine-treated or vehicle-control mothers, as described previously [9, 10, 13]. The slices (300–350 *μ*m) were cut transversely to the whole brain with a vibroslicer (Campden Instruments, Sileby, Loughborough, UK) and were instantly relocated to the artificial cerebrospinal fluid (aCSF) in an incubating chamber given with humidified 95% O_2_/5% CO_2_ gas at room temperature. For an incubation period of at least 60 minutes, a slice was moved to a submerged-type constant flow-recording chamber (volume 1.0 ml) refused with oxygenated aCSF (95% O_2_/5% CO_2_) at a rate of 1.0–1.5 ml/min at 30.0 ± 0.5°C.

### 2.3. Solutions and Drugs

The control aCSF consisted of (in mM) MgCl_2_ (1), NaCl (124), CaCl_2_ (2), NaH_2_PO_4_ (1.25), NaHCO_3_ (26), KCl (3.5), and D-glucose (10), pH 7.4. The solution used in the patch clamp electrode contained (in mM) CsCl (130), CaCl_2_ (1), EGTA (10), HEPES (10), NaCl (10), NaGTP (0.2), MgCl_2_ (3), Na_2_ATP (3), and QX-314 (5), pH 7.2 (using KOH). The osmolarity of the solutions was maintained at 305 ± 5 mOsm. Unless otherwise stated, the drug was dissolved in sterile water for a stock solution and was stored at −20°C. Slices were used for RNA preparation and reverse transcription-PCR and real-time PCR. All drugs in aCSF were primed instantly before each experiment from the frozen stocks and were given via bath application.

### 2.4. Real-Time Polymerase Chain Reaction (PCR)

RNA isolation, including DNase treatment, and synthesis of cDNA were performed with the Transcriptor First Strand cDNA Synthesis Kit RT-PCR System™ (Roche, Indianapolis, IN, USA), according to the suggestions of the manufacturer. Briefly, to synthesize cDNA, this study used 2 *μ*g of isolated total RNA and a combination of oligo-dT and arbitrary hexamer primers (20 *μ*l), provided in the kit. This study applied two-step real-time PCR with the LightCycler™ System (Roche, USA) and SYBR Green I dye for evaluating PCR. Preparing samples for real-time PCR, this study used FastStart DNA Master SYBR Green I (Roche, USA), following the suggestions of the manufacture. We used the following set of primers: PSD-95: forward primer (5′-ACG ACA AGA CCA AGG ACT GC), reverse primer (5′-TGG CCT TTA ACC TGG ACC AC); GluR1: forward primer (5′-ATG AGT TTG GGA ATC TCC ATT AT), reverse primer (5′-TCG CTG TGC CAT TCG TA); GluR2: forward primer (5′-CTA CCA ATG GGA CAA GTT CG), reverse primer (5′-CAG GAT TAC ACG CCG TT); GluR3: forward primer (5′-TGC AGT TAT ACA ACA CCA ACC), reverse primer (5′-TAG AAT CCA AAG ATA GCA TAC ACC C); GluR4: forward primer (5′-TGT AAC AGG ATT CCA GTT GGT AG), reverse primer (5′-CAT CAC CAG GAC TCC ATC ATA); NR1: forward primer (5′-TGT TCC GCG AGG CAG TAA A), reverse primer (5′-GAG TAG GCG GGT GGC TAA); NR2A: forward primer (5′-GCA TAT ACC ACG TAG GTG AAT C), reverse primer (5′-CTT CAT GGA ATT TGG AAT ATA GGC); NR2B: forward primer (5′-CGC ATC TGT CCA CCA TT), reverse primer (5′-GCA TCA GGA AAG CCT CG); NR2C: forward primer (5′-GTC CCT GTC CTG TTG TCA), reverse primer (5′-CGT TGC TTT AAT GTT CCA ATG C); NR2D: forward primer (5′-CCT TCT TCG CGG TCA TC), reverse primer (5′-CCG TGC CAA ACT TCA GAG); and 18S rRNA: forward primer (CCA GTA AGT GCG GGT CAT AA), reverse primer (TAG TCA AGT TCG ACC GTC TTC). There were 30–35 cycles for each program and 1.5 mM MgCl_2_. Amplification protocols included an initial step of DNA denaturation (4 min at 95°C), followed by 30–35 cycles each containing DNA denaturation for 30 seconds at 94°C, 1 minute at 62°C, and 1 minute at 72°C plus elongation for 7 minutes at 72°C. After amplification, 20 *μ*l of the PCR reaction mix was electrophoresed in an agarose gel (2%, wt/vol).

Each band was normalized against the corresponding loading control using a computer-assisted imaging analysis program. One-way ANOVA followed by post hoc Newman-Keuls tests was used for statistical difference in this study.

## 3. Results

### 3.1. Nucleus Accumbens

**Figure 1:**
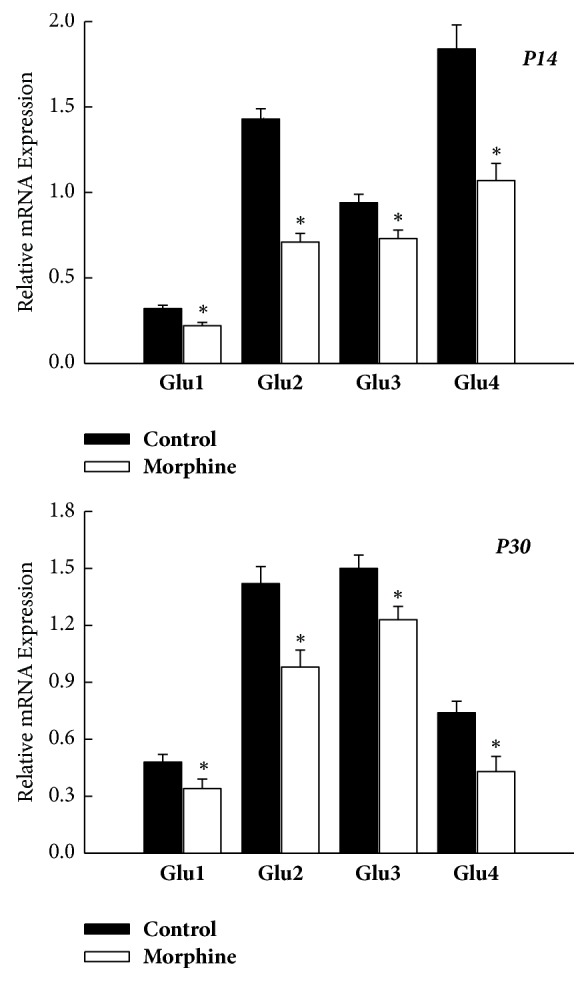
Prenatal morphine exposure resulted in a persistent decrease of the mRNA expression of AMPAR subunit (GluR1–4) within NAc of rat offspring, as assessed on P14 and P30, respectively. ^*∗*^*P* < 0.05 as compared with the vehicle-control group.

The AMPAR is heteromeric complex of four subunits, namely, GluR1-GluR4, respectively [7]. At P14 in nucleus accumbens, the mRNA level of Glu 1 was 0.22 ± 0.02 in prenatal morphine group and 0.32  ±  0.02 in control group. The mRNA level of Glu 2 was 0.7 ± 0.05 in prenatal morphine group and 1.43  ±  0.06 in control group. The mRNA level of Glu 3 was 0.73 ± 0.05 in prenatal morphine group and 0.94 ± 0.06 in control group. The mRNA level of Glu 4 was 1.07 ± 0.07 in prenatal morphine group and 1.94 ± 0.1 in control group. At P30 in nucleus accumbens, the mRNA level of Glu 1 was 0.34 ± 0.05 in prenatal morphine group and 0.48 ± 0.03 in control group. The mRNA level of Glu 2 was 0.92 ± 0.09 in prenatal morphine group and 1.42 ± 0.09 in control group. The mRNA level of Glu 3 was 1.2 ± 0.05 in prenatal morphine group and 1.5 ± 0.07 in control group. The mRNA level of Glu 4 was 0.41 ± 0.08 in prenatal morphine group and 0.74 ± 0.06 in control group. In brief, at the same age, the relative mRNA levels of the AMPAR subunit (GluR1-4) within NAc were all markedly decreased in the prenatal morphine group at P14 (*P* < 0.05, *n* = 8 animals, Figure 1) and P30 (*P* < 0.05, *n* = 8 animals, Figure 1), as compared with the vehicle-control group. Prenatal morphine exposure exerted a long-term downregulation on the AMPAR subunit (GluR1-4) through the transcription mechanisms within NAc, in a differential change of decreased magnitudes at P14 and P30. Furthermore, the greatest decrease in downregulation of gene expression of GluR2 and GluR4 was observed at P14 but not at P30.

**Figure 2:**
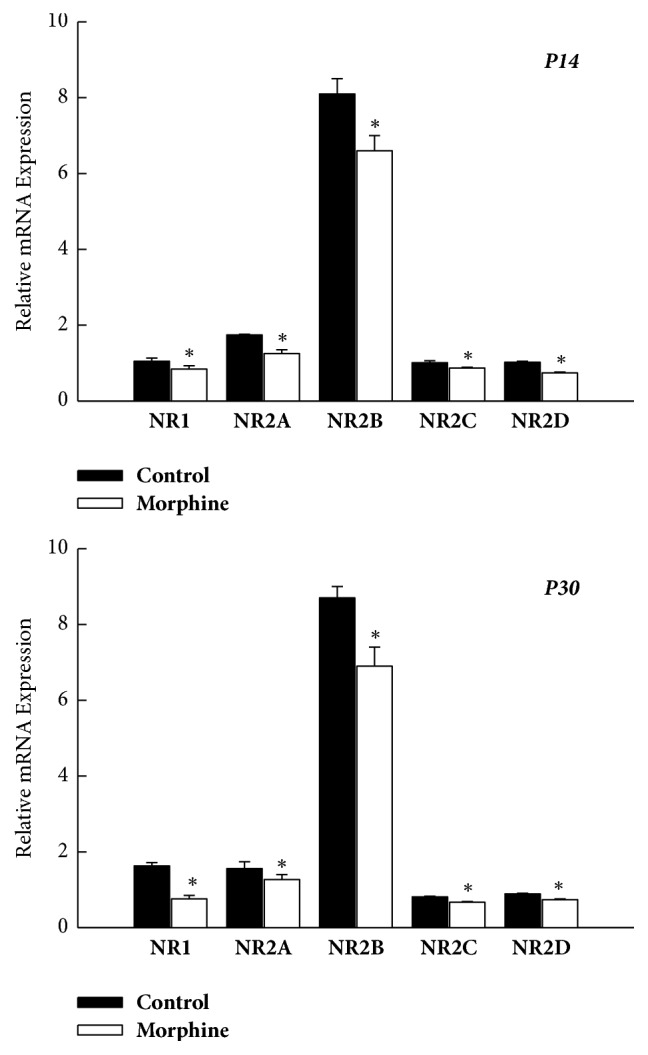
Prenatal morphine exposure led to a persistent decrease in the mRNA expression of NMDAR subunit (NR1 and NR2A–D) within NAc of rat offspring, as assessed on P14 and P30, respectively. ^*∗*^*P* < 0.05 as compared with the vehicle-control group.

The NMDAR acts as a heterometric complex containing a NR1 subunit with one or more of the four different NR2 subunits (NR2A-D) [14]. The relative mRNA levels of the NMDAR subunit (NR1, NR2A-D) within NAc were all significantly decreased in the prenatal morphine group at both P14 (*P* < 0.05, *n* = 8 animals, Figure 2) and P30 (*P* < 0.05, *n* = 8 animals, Figure 2), as compared with the vehicle-control group. The expressions of all units of the NMDAR subunit were all downregulated by prenatal morphine exposure, and these changes exhibited a long-lasting manner.

### 3.2. Ventral Tegmental Area

**Figure 3:**
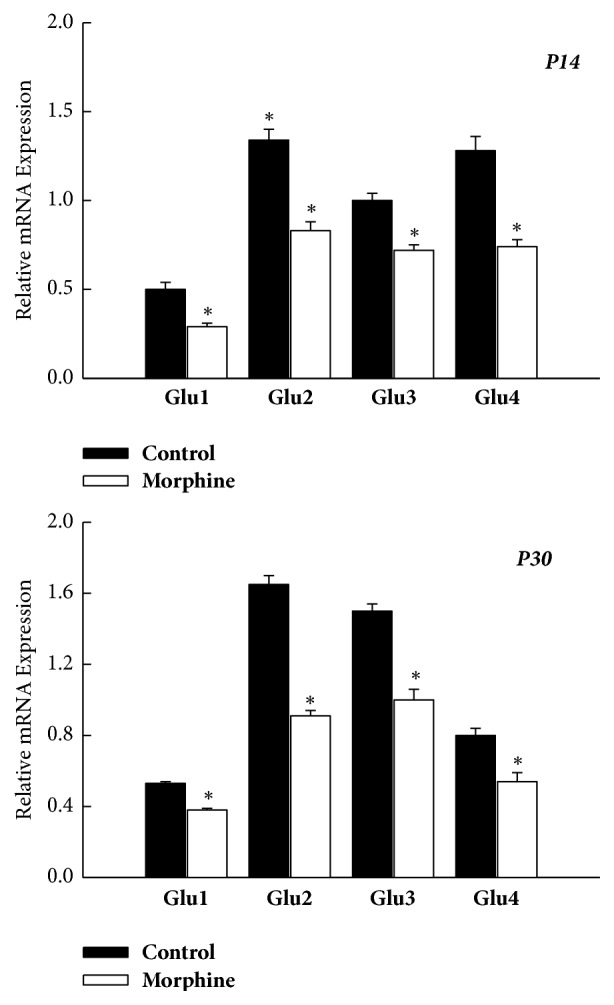
Prenatal morphine exposure induced a long-lasting decrease in the mRNA expression of AMPAR subunit (GluR1–4) within VTA of rat offspring, as assessed on P14 and P30, respectively. ^*∗*^*P* < 0.05 as compared with the vehicle-control group.

The relative mRNA levels of all the AMPAR subunit (GluR1–4) within VTA were all significantly decreased in the prenatal morphine group, as compared with the vehicle-control group at the same age (*P* < 0.05, *n* = 8 animals for each time point, Figure 3). Apparently, prenatal morphine exposure also exerted a long-term downregulation on the AMPAR subunit (GluR1–4) through the transcription mechanisms within ventral tegmental area.

**Figure 4:**
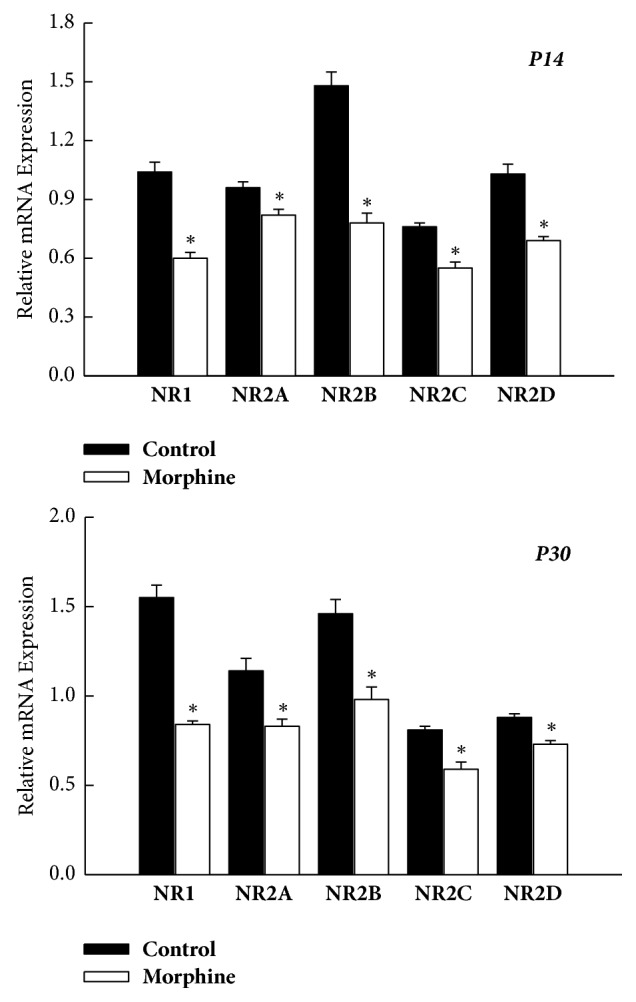
Prenatal morphine exposure resulted in a persistent decrease in the mRNA expression of NMDAR subunit (NR1 and NR2A–D) within VTA of rat offspring, as assessed on P14 and P30, respectively. ^*∗*^*P* < 0.05 as compared with the vehicle-control group.

Simultaneously, the relative mRNA levels of the NMDAR subunit (NR1, NR2A–D) within VTA were also revealed to have significantly decreased in the prenatal morphine group at P14 (*P* < 0.05, *n* = 8 animals, Figure 4) and P30 (*P* < 0.05, *n* = 8 animals, Figure 4), as compared with the vehicle-control group. Apparently, prenatal morphine exposure exerted a long-term downregulation on the NMDAR subunit (NR1, NR2A–D) through the transcription mechanisms, not only within nucleus accumbens but also within ventral tegmental area.

### 3.3. Prefrontal Cortex

**Figure 5:**
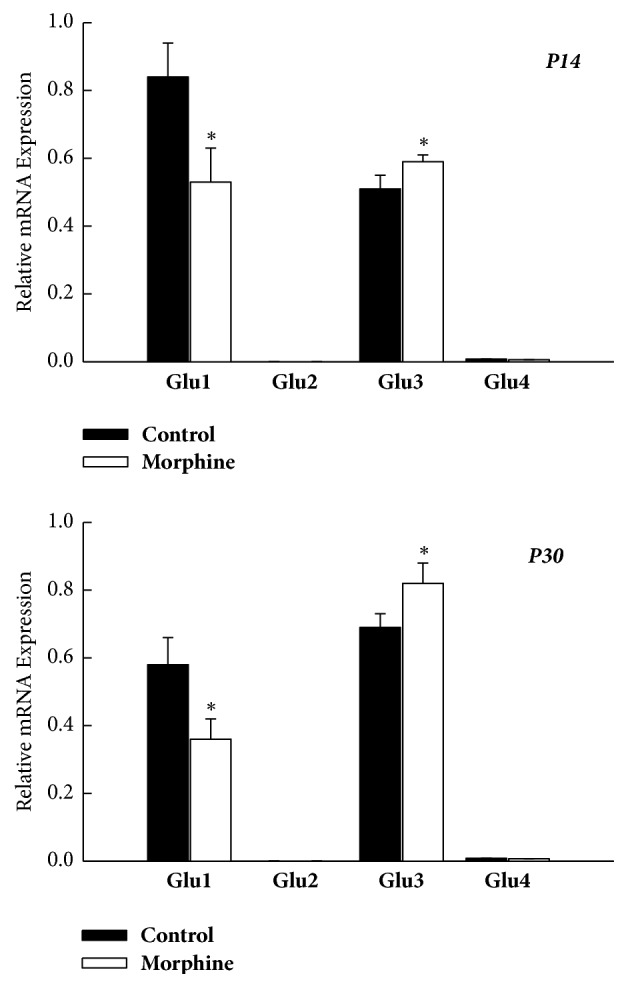
Summary of mRNA levels of the AMPAR subunit (GluR1–4) in the PFC of rat offspring, as assessed on P14 and P30, respectively. ^*∗*^*P* < 0.05 as compared with the vehicle-control group.

As shown in Figure 5, the mRNA expression in the AMPAR subunit was all declined within PFC of rat offspring subjected to prenatal morphine exposure. The relative mRNA expressions of GluR2 and GluR4 were barely detected within PFC at both P14 and P30 without any statistical difference (*P* > 0.05, *n* = 8 animals, resp., Figure 5). While the mRNA level of GluR1 was decreased, the mRNA level of GluR3 was increased in prenatal-morphine-exposure group compared to vehicle-control group at P14 (*P* < 0.05, *n* = 8 animals, Figure 5) and P30 (*P* < 0.05, *n* = 8 animals, Figure 5). The results here showed that not all the gene expression exhibited the same regulation pattern in rat offspring born to morphine-addicted mothers. Furthermore, GluR1 showed downregulation, while GluR3 exhibited upregulation, concurrently. GluR2 and GluR4 showed no specific change in PFC, as compared to the vehicle-control group.

**Figure 6:**
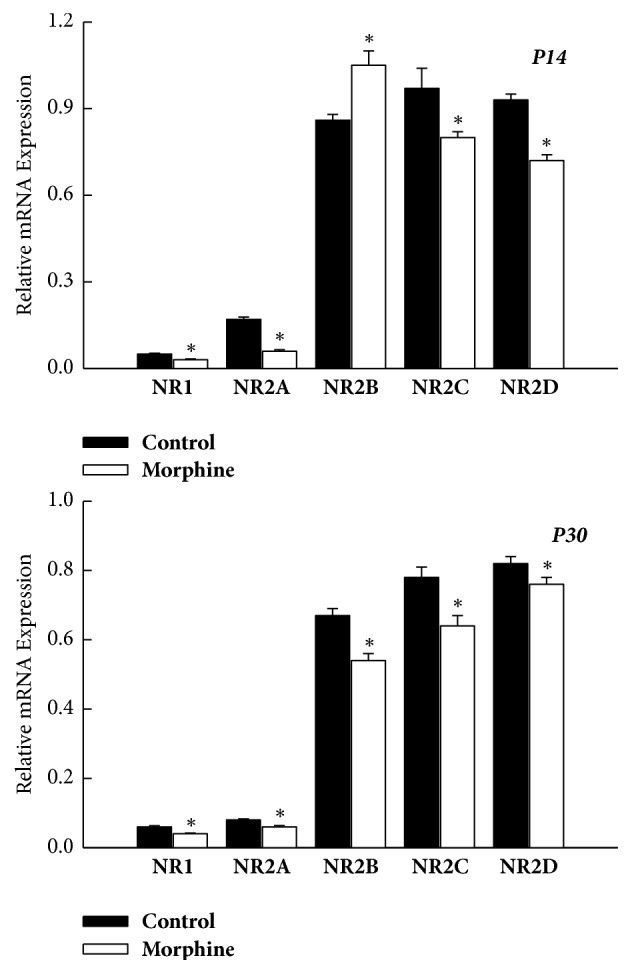
Summary of mRNA expression levels of the AMPAR subunit (GluR1, GluR2, GluR3, and GluR4) in PFC of rat offspring, as assessed on P14 and P30, respectively. The differential change of mRNA levels of the AMPAR subunit was noticed in rat offspring. It was worth noting that mRNA levels of GluR1 were decreased, but mRNA levels of GluR3 were increased in rat offspring in prenatal morphine exposure group, as compared to vehicle-control group. The mRNA level of GluR2 and GluR4 did not show significant difference. ^*∗*^*P* < 0.05 as compared with the vehicle-control group.

However, not all the relative mRNA levels of the NMDAR subunit (NR1, NR2A-D) within PFC were significantly decreased in the prenatal morphine group at P14 and P30. The relative mRNA levels of NR1, NR2A, NR2C, and NR2D were all declined in PFC at P14 (*P* < 0.05, *n* = 8 animals, Figure 6) and P30 (*P* < 0.05, *n* = 8 animals, Figure 6), as compared with the vehicle-control group at the same age (*n* = 8 animals for each time point). Interestingly, at P14, the mRNA level of NR2B was significantly increased in PFC of rat offspring subjected to prenatal morphine exposure. In contrast, the mRNA level of NR2B was significantly declined in PFC, as compared to the vehicle-control group.

### 3.4. Postsynaptic Density Protein 95

The postsynaptic density 95 (PSD-95), one of PDZ domain-containing proteins, interacted with the NMDAR and AMPAR. Primarily, the PDS-95 and NMDAR form the synaptic complex that is basically concerned with the regulation of synaptogenesis, synaptic plasticity, neuronal survival, learning, memory, neuronal differentiation, and maturation [6]. The PSD-95 also takes part in the regulation of synaptic AMPA receptors. Therefore, we further examined whether the gene expression in PSD-95 would be changed in rat offspring from morphine-addicted mothers.

**Figure 7:**
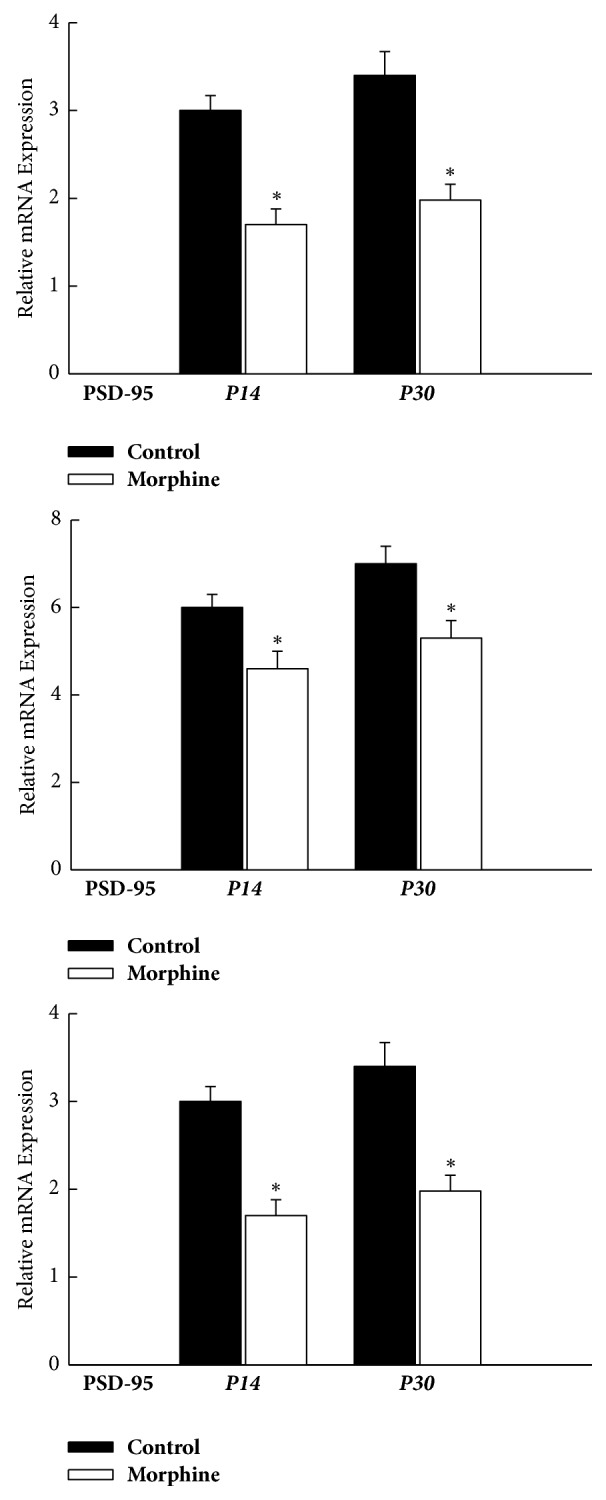
Concomitant decreases in mRNA expression levels of PSD-95 within the mesocorticolimbic system of rat offspring, as assessed on P14 and P30, respectively. ^*∗*^*P* < 0.05 as compared with the vehicle-control group. NAc: nucleus accumbens; VTA: ventral tegmental area; PFC: prefrontal cortex.

The mRNA expressions of PSD-95 were all decreased significantly within NAc, VTA, and PFC concomitantly, compared with the vehicle-control group at P14 (*P* < 0.05, *n* = 8 animals, Figure 7) and P30 (*P* < 0.05, *n* = 8 animals, Figure 7). The results suggest that mRNA expression of PSD-95 was also downregulated within mesocorticolimbic system in the rat offspring from morphine-addicted mothers in a long-lasting manner.

## 4. Discussion

This study has characterized the prenatal-morphine-exposure results in a long-lasting alteration in mRNA expression of the AMPAR, NMDAR, and PSD-95 within the mesocorticolimbic system, which is responsible for drug reward and dependence. In addition, such prenatal morphine exposure in offspring, regarding the gene expression of ionotropic glutamate receptor subunit and PSD-95, would appear not only during the infant age [15] (e.g., P14) but also in the adolescent age [15] (e.g., P30).

Prenatal exposure to opiates not only causes intrauterine growth retardation, higher rate of intrauterine death, and opiate withdraw symptoms after birth but also would lead to long-term neurobiological alterations, including the modifications in seizure susceptibility, sensitivity in morphine-induced analgesia, and social behavior such as inattention, hyperactivity, aggression, or drug craving behavior [4]. Nevertheless, it is difficult to accurately assess the effects of prenatal drug exposure on human brain in clinical cohort. Therefore, animal studies are required. Prenatal morphine exposure might lead to increased activity and/or hypersensitivity of the opiate system and result in the offspring higher risk of opiated drug addiction [16]. Besides, prenatal morphine exposure raises the reinforcing effects of opiates by self-administration in animal model [17]. The mesolimbic system, including nucleus accumbens, ventral tegmental area, and prefrontal cortex, is involved in the neural circuitry underlying cue elicited drug-seeking behavior [18]. Therefore, we investigated these three areas to evaluate the possible effect of perinatal morphine exposure. In addition, it was generally believed that glutamatergic input onto the mesolimbic system and adaptive change in glutamate receptor appear to modulate the strengthening properties of abusing drugs and reward-dependent learning process. Indeed, the inotropic glutamatergic system, including the AMPAR and NMDAR, within the mesolimbic system has been proposed to play an important role not only in synaptic plasticity but also in the development of addictive behavior.

According to previous study, Russell et al. found that the presence of GluR1 within NAc was increased in a morphine-addicted rat model [7]; however, in Mickiewicz and Napier's study, Glu 1 and Glu 2 expressions were unchanged under repeated morphine exposure [19]. In contrast, in our experimental model, we found that all the AMPAR subunits within NAc were all decreased. In addition, Murray et al. found that the expressions of NR1 and NR2A of the NMDAR were enhanced in NAc of morphine-dependent rats [20]. In this study, the gene expression of NMDAR subunit was decreased in VTA of rat offspring subjected to prenatal morphine exposure. Lane et al. highlighted that GluR1 of the AMPAR was increased [21]. Interestingly, in our study, the gene expression in all the AMPAR subunit was decreased in this prenatal-morphine-exposure rat model. In PFC, previous study found that the repeated morphine exposure would decrease expression of GluR1 without affecting GluR2 subunit [19]. Regarding the NMDAR in PFC, Murray et al. found taht the expressions of NR1 and NR2A were decreased, but NR2B was unchanged in morphine-dependent rats [20]. In this study, the gene expressions of GluR1, NR2A, and PSD-95 were all decreased, and NR2B was increased. Similarly, the PSD-95 expression is modulated by epigenetic regulation under morphine exposure [22] and so did our study that the expressions of PSD-95 were all attenuated under prenatal morphine exposure in NAc, VTA, and PFC. It was likely that prenatal morphine exposure resulted in a long-lasting modulation of the AMPA/NMDAR subunit in NAc, VTA, and PFC. Previously, Pertschuk et al. indicated that the amount of the opiate receptor in the developing brain was more than the adult; therefore, these neonates are more sensitive to the prenatal exposure [23]. And, in humans, opiates would rapidly cross the placenta. Little is known about the effect of prenatal opiate exposure and the possible modulation and damage of prenatal exposure. This study provides additional information on the transplacental effect of morphine on the synapse over the mesolimbic system.

In this study, the concurrent change of the AMPAR, NMDAR, and PSD-95 not only affected as early as infant period (e.g., P14), but also the modulation would be long-lasting to adolescent period (e.g., P30). We found that perinatal morphine exposure might lead to long-lasting attenuated NR1 and Glu 1 mRNA expression within NAc, VTA, and PFC in the offspring. Here, this study suggests that synaptic plasticity would be modified accompanied with the gene expression change of inotropic glutamate receptor subunits, concomitantly. Our previous study revealed that prenatal administration of morphine would alter the phosphorylation and synaptic plasticity was mediated by glutamatergic transmission over hippocampal area of cognitive-deficient rat offspring [9–13]. These findings all support the hypothesis of a dysregulation of the glutamate system and the corresponding modification of synaptic plasticity in mesolimbic system in the development of addiction. Such modifications seen in this study might correlate with the possible brain damage in the human and hint at the long-term neurobiological alterations with behavior sensitization, inattention, and motor and cognitive impairments.

AMPAR is composed of four subunits, and GluR1 to GluR4 participate in the channel formation. These subunits combine in different and various combinations* in vitro*; however, in central nervous system, GluR1 and GluR2 subunits are ubiquitously expressed. GluR2 within NAc was considered to be associated with relapse-provoking reward during prolonged abstinence of opiate [24]. During development, GluR2 was low expressed in fetus central nervous system [25, 26]. Later after birth, AMPAR would change from the GluR2-lacking to GluR2-containing AMPAR in rat central nervous system [27, 28]. In our study, we demonstrated that the level of GluR2 was abundant in NAc and VTA at both P14 and P30. However, in PFC, the mRNA expressions of GluR2 and GluR4 were barely detected at P14 and P30. The magnitude of change of GluR2 in rat offspring subjected to prenatal morphine exposure was also significant in NAc and VTA. It was likely that GluR2 might be more vulnerable to prenatal morphine exposure.

NMDAR is a heterotetrameric ionotropic receptor. Seven NMDAR subunits have been identified: NR1, NR2 (A–D), and NR3 (A, B). Native NMDAR are mostly composed of two NR1 and two NR2 subunits. The type of the NR2 critically determined biophysical and pharmacological properties of the NMDAR [26], especially NR2A and NR2B, which are responsible for long-term potentiation. Besides, the four NR2 subunits also show a different distribution in the central nervous system, and the expression would change during development. NR2A, NR2B, and NR2C are abundant during early development stages. In our study, we observed that the gene expression of NR2B subunits was the greatest among all subunits within NAc, VTA, and PFC at P14. Both Kao et al. and Shen et al. found that NR2B might be responsible in morphine rewarding effect and seeking effect, especially in NAc in the adult rat with chronic use of morphine, not in VTA [29, 30]. Taken together, this study further provided information that the mRNA expression of NR2B was persistently attenuated the most in NAc at P14 and P30 in rat offspring from morphine-addicted mothers. However, in PFC and VTA, the magnitude of the changes in mRNA expression of NR2B was not relatively robust, although they still showed statistical significance at P14 and P30. In PFC, we observed that the mRNA expression of NR2B was significantly increased at P14 but markedly declined at P30. Taken together, these findings suggest that a long-lasting, differential alteration in NR2B of the NMDAR may play a role for the morphine-induced rewarding effect and craving behavior, as a function of ages.

The synaptic complex of PSD-95 with the NMDAR and AMPAR regulates synaptogenesis, synaptic plasticity, ion-channel functioning, and learning and memory function. It has been shown that synaptic complex of PSD-95 with the NMDAR and AMPAR subunit is dynamically changed in regulation and modulation in animals suffering with experimentally induced insults such as perinatal hypoxia-ischemia [31]. Previously, we have shown that prenatal morphine exposure attenuated the PSD-95 and NMDAR interaction within hippocampal CA1 region in rat offspring [31]. In this study, we further found that the PSD-95 mRNA expression was persistently reduced under the insult of perinatal morphine exposure within NAc, VTA, and PFC at both P14 and P30.

AMPA receptors in nucleus accumbens are related to rewarding and seeking behavior in opiate addiction [24]. NMDA receptor might be involved in the memory formation and rewarding behaviors [32]. In observation, administering NMDA receptor antagonist in human, it seemed to reduce opiate reward but did not alter reinforcement [33]. Rodents exposed to opiates in uterus may become more sensitive to drug reward in the adulthood [34]. Therefore, the modification of AMPA receptor and NMDA receptor under prenatal opiate exposure might result in behavior problem in the future.

According to previous study, the sex differences after prenatal opiate exposure mainly depend on the presence or absence of sex hormone [35]. However, in our study, we discussed the alternation in the rat offspring earlier before developing the reproductive system. Besides, in the first few days after birth, it was not easy to identify the sex of pups. Therefore, in our study, we included both male and female rats in the study and there were no statistical differences between the groups. In the future, some new experiment might be designed to compare the gender effect in such young age.

## 5. Conclusion

In summary, this study provides the additional evidence that prenatal morphine exposure could result in a concomitant, long-lasting, differential change of mRNA level of the AMPAR/NMDAR and PSD-95 within mesocorticolimbic system. We also suggested that prenatal morphine exposure persistently altered gene expression in PSD-95 within such neuronal circuitry in which NAc, VTA, and PFC are responsible for the integration of drug-craving behavior in mammals. Further experiments are needed to unveil mechanisms causing such variable alterations in gene expression of offspring born to morphine-addicted mothers.

## Data Availability

All data arising from this study are contained within the manuscript.
